# Author Correction: Spatial fidelity of workers predicts collective response to disturbance in a social insect

**DOI:** 10.1038/s41467-018-04598-7

**Published:** 2018-05-31

**Authors:** James D. Crall, Nick Gravish, Andrew M. Mountcastle, Sarah D. Kocher, Robert L. Oppenheimer, Naomi E. Pierce, Stacey A. Combes

**Affiliations:** 1 Department of Organismic and Evolutionary Biology, Harvard University, 26 Oxford St., Cambridge, MA 02143 USA; 2 Mechanical and Aerospace Engineering, University of California San Diego, Engineer Ln, San Diego, CA 92161 USA; 3 Department of Biology, Bates College, 2 Andrews Road, Lewiston, ME 04240 USA; 4 Lewis-Sigler Institute for Integrative Genomics, Princeton University, Princeton, NJ 08540 USA; 5 Department of Biological Sciences, University of New Hampshire, 105 Main St., Durham, NH 03824 USA; 6 Department of Neurobiology, Physiology, and Behavior, University of California Davis, Davis, CA 95616 USA

Correction to: *Nature Communications* 10.1038/s41467-018-03561-w, published online 03 April 2018

The original version of the Article contained incorrect citation information in reference 67. The reference should read “Russell, A. L., Morrison, S. J., Moschonas, E. H. & Papaj, D. R. Patterns of pollen and nectar foraging specialization by bumblebees over multiple timescales using RFID. *Sci. Rep*. **7**, 1–13 (2017).” This error has now been corrected in the PDF and HTML versions of the Article.

