# Supplementary material for: Spatial fidelity of workers predicts collective response to disturbance in a social insect
Source: Nat Commun. 2018 Apr 3;9:1201. doi: 10.1038/s41467-018-03561-w (PMC5882771; doi:10.1038/s41467-018-03561-w)
Supplement: Supplementary file 1 — Supplementary Information(PDF 1399 kb) [file 41467_2018_3561_MOESM1_ESM.pdf]

Supplementary Information for

Crall et al., Spatial fidelity of workers predicts collective response to disturbance in a social insect

Supplementary Table 1. Abbreviations, description, and principle component loadings of all 23 nest behavior metrics used in nest behavior PCA.

Abbreviation	Description	PC1 loading	PC2 loading
$Cor_{spat,frgr}$	Spatial correlation strength to foragers	0.228	0.238
$Cor_{spat, all}$	Spatial correlation strength to all nestmates	0.229	0.267
$Cor_{spat, nfrgr}$	Spatial correlation strength to non-foragers	0.211	0.268
$R_{int,BR}$	Brood physical interaction rate	0.228	0.22
$D_{BR,nearest}$	Median distance to brood (nearest)	-0.239	-0.216
$P_{BR, day}$	Proportion of nest time on brood (day)	0.052	0.234
$P_{BR, night}$	Proportion of nest time on brood (night)	0.072	0.215
$R_{int,WP}$	Waxpot interaction rate	0.237	-0.072
$D_{WP,nearest}$	Median distance to waxpots (nearest)	-0.258	0.02
$D_{BR,all}$	Median distance to brood (all)	-0.289	0.005
$D_{WP,all}$	Median distance to waxpots (all)	-0.285	0.062
$D_{cent, night}$	Mean instantaneous distance to social center (night)	-0.304	0.017
$D_{cent, day}$	Mean instantaneous distance to social center (day)	-0.305	0.077
RA_{50}	Nest area home range (50%)	-0.275	0.174
RA_{90}	Nest area home range (90%)	-0.295	0.179
$P_{active,day}$	Portion of time active (day)	0.021	0.246
$Circ_{str}$	Circadian activity scope	-0.052	-0.18
$P_{active,night}$	Portion of time active (night)	0.058	0.328
$Speed_{mov}$	Moving speed	-0.125	0.184
Dev_{speed}	Standard deviation of speed	-0.068	0.217
$Disp_{bw,ts}$	Between trial displacement within nest	-0.185	0.259
$Disp_{nest,occ}$	Nest occupancy dispersion index	0.087	-0.299
$Gini_{nest\ occ}$	Nest occupancy Gini	0.175	-0.299

Supplementary Table 2. Cross-tabulation of behavioral sequences identified manually and by automated classification. Behavioral sequences (n = 857) were manually classified from videos following published ethograms[60,61], then aggregated into three categories: Nursing (N), Patrolling (P+B), and Inactive (In), corresponding with the categories generated by the automated behavioral annotation. The same sequences were then automatically classified as described in the main text. Automated behavioral annotation corresponded correctly with behavioral categories 86% of the time, significantly different from expectation (df = 24, Chi-squared = 841, $p < 10^{-100}$).

			Automatic annotation		
			**Nursing**	**Patrolling**	**Inactive**
Manual annotation	**Nursing**	*Anchoring*	**10**	1	2
		Drinking Honey	**5**	0	0
		Incubating	**62**	0	0
		Inspecting	**310**	5	3
		Scraping Wax	**11**	2	1
		Working Honey Pot	**70**	0	0
		Laying Eggs	**19**	0	0
		Probing Honey Pot	**30**	0	0
	**Patrolling**	*Buzzing*	4	**23**	9
		Patrolling	4	**26**	26
	**Inactivity**	*Resting*	9	11	**73**
		Inactivity	6	3	**70**
		Perching	21	12	**29**

Summary

		Automatic			
		**Nursing**	**Patrolling**	**Inactive**	**Total**
Manual	**Nursing**	**517**	8	6	531
	**Patrolling**	8	**49**	35	92
	**Inactivity**	36	26	**172**	234
	**Total**	561	83	213	**857**
	**Correct annotation rate**	***0.92***	***0.59***	***0.81***	***0.86***

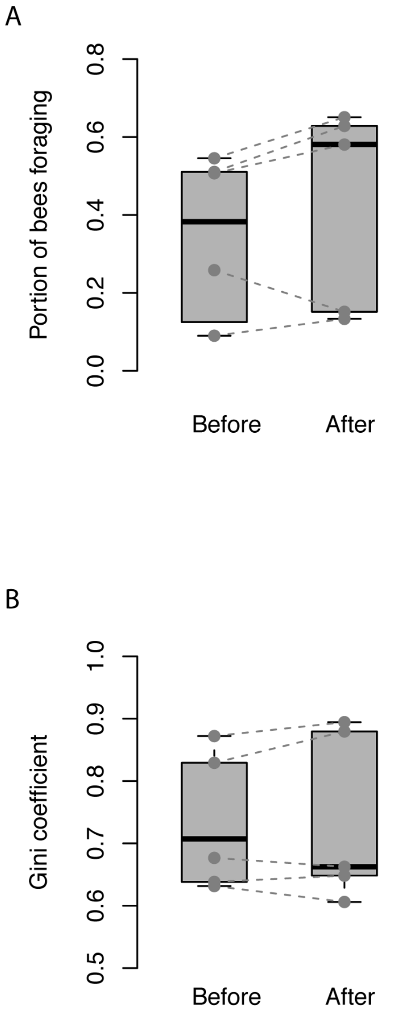

Supplemenetary Figure 1. Foraging in *B. impatiens* colonies (n = 5) before and after exposure to a non-foraging manipulation. The manipulation consisted of temporary removal (~5 hours) and separate housing of workers for non-invasive metabolic measurements, the results of which are not reported here. (A) Portion of bees foraging and (B) Gini coefficient of foraging activity in the three days before (left) and three days after (right) the manipulation, the same time periods examined for forager removal experiments. Grey dashed lines connect values from the same colonies before and after treatment. Boxplots show the median and inter-quartile range (IQR), while whiskers depict the data range. There were no significant differences before and after manipulation for either portion of bees foraging (paired t-test, $t = 1.15$, $df = 4$, $p = 0.31$) or Gini coefficient of foraging activity (paired t-test, $t = 0.63$, $df = 4$, $p = 0.56$).

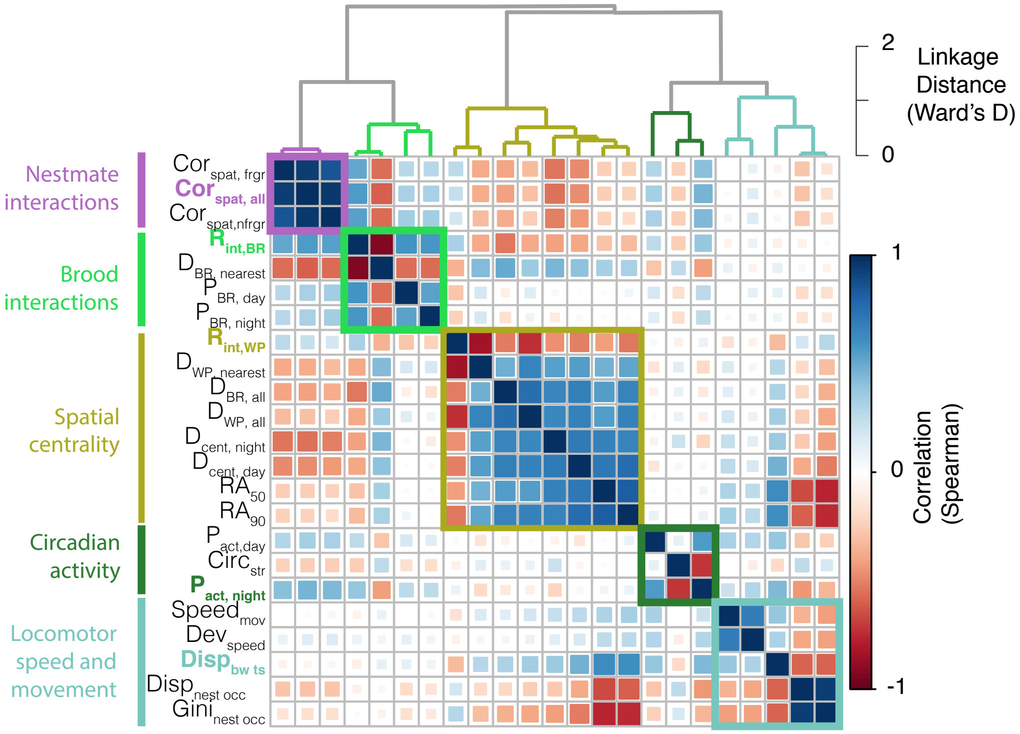

Supplementary Figure 2. Pairwise correlation matrix between individual metrics of behavior (see Supplementary Table 1 for variable descriptions), with correlation direction and strength indicated by color. Colored labels indicate clusters of related variables based on a hierarchical clustering analysis. Bold color labels indicate individual variables used in subsequent analyses (Supplementary Figure 3).

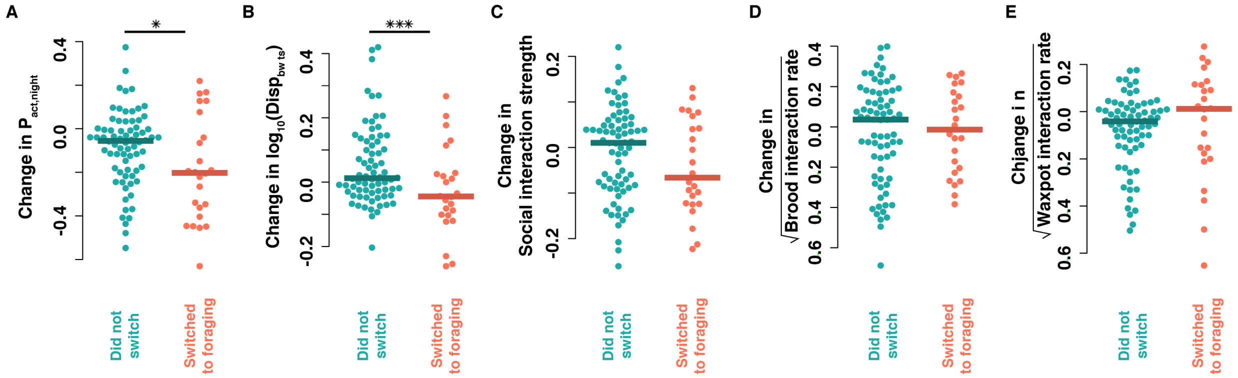

Supplementary Figure 3. Switching to foraging changes components of locomotor activity, but not spatial centrality. Beeswarm plots of change in five nest behavior metrics (one from each cluster in Supplementary Figure 2) after disturbance by foraging status after disturbance, among bees that had performed no foraging before disturbance. (A) Proportion of time active; $t = -2.26$, $df = 92.8$, $p = .026$, (B) Mean displacement between timesteps; $t = 3.64$, $df = 92.7$, $p = 0.0004$. (C) Social interaction strength; $t = -1.16$, $df = 92.8$, $p = 0.25$. (D) Brood interaction rate; $t = 0.78$, $df = 92.6$, $p = 0.44$, and (E) Waxpot interaction rate; $t = 0.97$, $df = 93.5$, $p = 0.33$. Statistics show results of linear mixed effects models with foraging status in the three days after disturbance as a fixed effect and colony as a random effect, analyzing only workers who performed no foraging activity in the three days before disturbance.

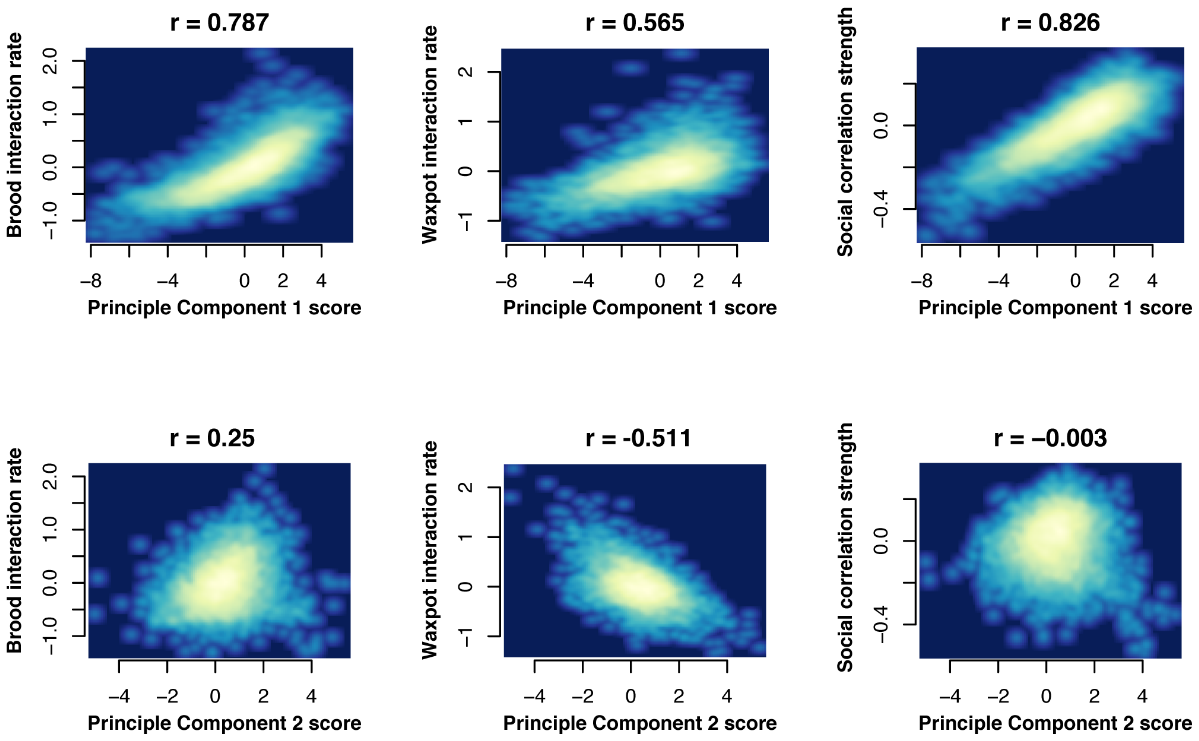

Supplementary Figure 4. Pairwise correlations between PC1 scores (top row) and PC2 scores (bottom row), with brood interaction rate (left column), waxpot interaction rate (middle column), and social interaction strength (right column). Colors show relative density of points (n = 1132) from blue to yellow.

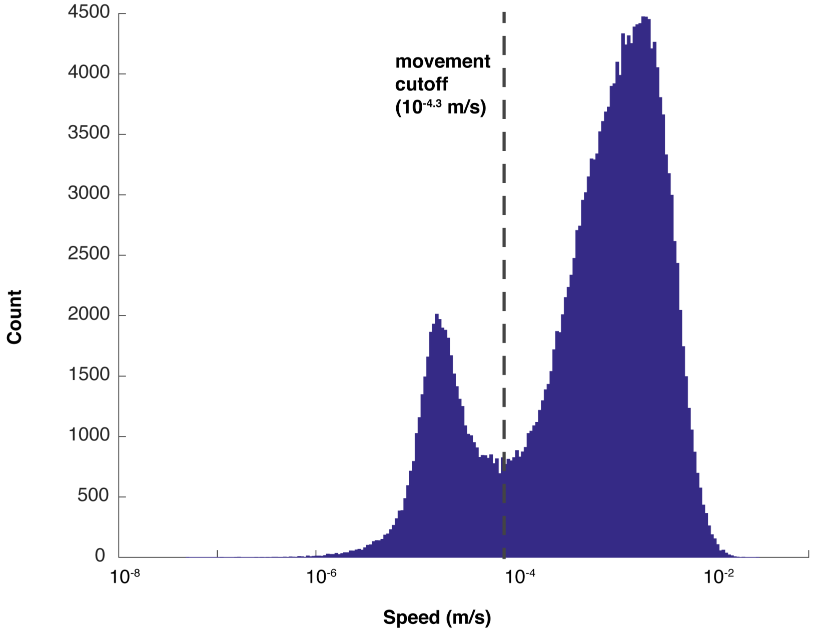

Supplementary Figure 5. Bimodal distribution of instantaneous movement speeds within bumblebee nests, shown for a single representative colony.

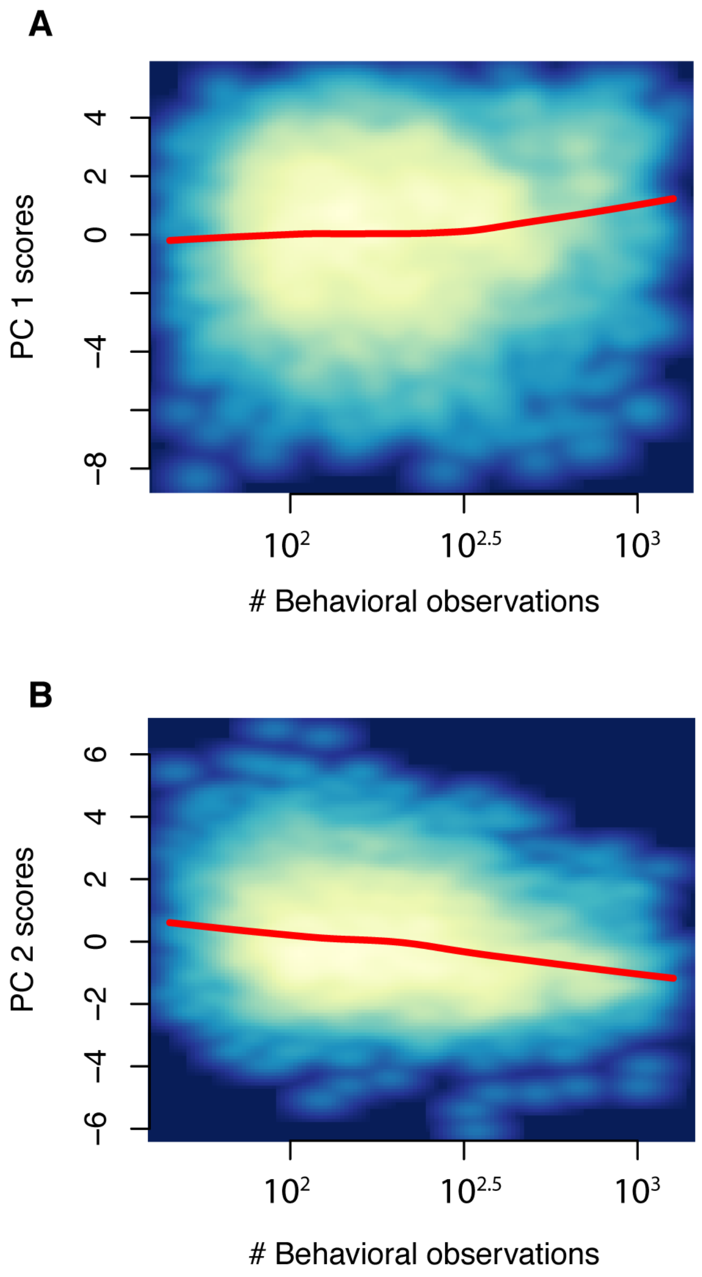

Supplementary Figure 6. Relationship between the number of behavioral observations and (A) PC1 and (B) PC2 scores. Red lines show Lowess smoothing of local trend lines, and colors show relative density of points (n = 1132) from blue to yellow.
