# Supplementary material for: Spatial fidelity of workers predicts collective response to disturbance in a social insect
Source: Nat Commun. 2018 Apr 3;9:1201. doi: 10.1038/s41467-018-03561-w (PMC5882771; doi:10.1038/s41467-018-03561-w)
Supplement: Supplementary file 3 — Description of Additional Supplementary Files(PDF 171 kb) [file 41467_2018_3561_MOESM3_ESM.pdf]

Description of Supplementary Files

File Name: Supplementary Movie 1
Description: Tracking of individual bumblebees within the nest. Dark green shows raw, and light green interpolated, locations of individual bees.

File Name: Supplementary Movie 2
Description: Tracking of individual foraging activity. Outwardly oriented bees are shown in green, and inwardly oriented bees in red.

File Name: Supplementary Movie 3
Description: Spatial mapping of worker locations and brood elements in bumblebee colonies. Large transparent circles show mapped nest elements (yellow and red show larvae and pupae, respectively, and green, purple, and blue show empty waxpots, full waxpots, and wax coverings, respectively). Small transparent red dots show bee coordinates over a single behavioral sequence. Tracks of individual bees for each frame are overlaid with open orange or blue circles if the bees were located on the brood or waxpots, respectively.
